# The Price of digital sovereignty: how Data Self-Efficacy and perceived foreign data dependency drive citizen acceptance of costly cross-border data protection

**DOI:** 10.3389/fpsyg.2026.1889373

**Published:** 2026-10-02

**Authors:** Haowen Song, Meichao Zhao, Yuhan Gao, Haowei Wang, Qi Cao

**Affiliations:** 1 Department of International Relations, Graduate School, Yonsei University, Wonju, Gangwon State, Republic of Korea; 2 Department of Business Administration, Graduate School, Sejong University, Seoul, Republic of Korea; 3 School of Advanced Technology, Xi'an Jiaotong-Liverpool University, Suzhou, Jiangsu, China; 4 Department of Management and Marketing, Faculty of Business Administration, University of Macau, Macau, Macao SAR, China; 5 Department of East Asian Studies, Graduate School of International Studies, Hanyang University, Seoul, Republic of Korea

**Keywords:** cross-border data flow, Data Self-Efficacy, digital sovereignty, PLS-SEM, policy acceptance, costly policy support

## Abstract

Citizens increasingly endorse data protection policies whose costs are tangible and individual while their benefits are diffuse, collective, and geopolitical—a pattern poorly accommodated by privacy calculus models and largely overlooked in the international relations literature on data statecraft. Bridging these traditions, this study advances a microfoundational account in which Perceived Foreign Data Dependency operates as a cognitive bridge between macro-level geopolitical conditions and individual acceptance of costly data protection. Two parallel channels are specified—a risk pathway through Perceived Cross-border Data Risk and an opportunity pathway through Tolerance for Cross-Border Data Openness—with Data Self-Efficacy positioned as a moderator arbitrating substitute and complement accounts of citizen capability. Survey evidence from South Korean adults, analysed via partial least squares structural equation modelling, indicates that the two channels operate as complementary rather than competing mediators. Contrary to the discriminating-citizen prediction, higher digital capability does not partition legitimate openness benefits from illegitimate dependency risks; instead, it sharpens an integrative response in which registered dependency triggers a concurrent downward revision of the normative legitimacy of cross-border openness. This reversed-direction moderation is reconceptualised as the Enlightenment-Vigilance hypothesis, contributing a cognitive-bridge linkage between the geopolitics-of-data and privacy decision literatures and reframing the long-standing substitute-versus-complement debate over digital capability.

## 1 Introduction

Citizens across diverse political systems increasingly inhabit an information environment in which the governance of personal data has become inseparable from cross-border flow, jurisdictional reach, and infrastructural reliance on foreign-controlled systems. From the Schrems II ruling (Case C-311/18, 2020) and the European Data Act to India’s Digital Personal Data Protection Act and the Republic of Korea’s K-Cloud program, data localization and sovereign data infrastructure have become salient features of contemporary policy discourse (Aaronson, 2019; Chander and Lê, 2015). This shift exemplifies what Farrell and Newman (2019) call the weaponization of economic interdependence, and it has accelerated markedly since 2023 as the EU AI Act (Regulation 2024/1689) and the global diffusion of generative AI extend the locus of data sovereignty from storage and transfer into model training, weight provenance, and inference-layer dependencies (Bradford, 2023; Floridi, 2020). Recent scholarship has accordingly reframed digital sovereignty as a multi-dimensional phenomenon spanning legal jurisdiction, infrastructural control, and epistemic asymmetry vis-a-vis foreign AI systems (Chung, 2026; Santaniello, 2025), reaching into the computational substrate of artificial intelligence itself (Akıllı and Shkurti Özdemir, 2025; Farrell and Newman, 2023).

What is striking, however, is not the policy turn itself but the apparent willingness of citizens to endorse it. Eurobarometer data show that 72% of European respondents express concern about losing control over their online information, and 56% support stronger national or regional safeguards even when these constrain cross-border services (European Commission, 2019); comparable patterns appear in East Asia and North America (Auxier et al., 2019; Park, 2013). This pattern sits uneasily with the dominant privacy calculus tradition (Dinev and Hart, 2006; Smith et al., 2011), which models data-related decisions as transactional risk–benefit assessments and presumes that individuals withdraw support when policies impose tangible personal costs. A growing body of post-2024 scholarship has begun to extend this tradition to generative AI services, documenting how users continue to weigh risks against personalization benefits and how perceived anthropomorphism raises privacy concerns and perceived benefits at the same time (Zhou and Tang, 2025), how AI-specific privacy awareness remains uneven (Dahabiyeh et al., 2026), and how heightened privacy concern can weaken rather than strengthen the translation of disclosure awareness into protective behavior (Mazhar et al., 2026). Yet these contributions, like the privacy paradox literature they extend (Acquisti et al., 2015; Kokolakis, 2017), remain anchored at the individual-service interface. Why, then, would individuals express willingness to bear higher prices, reduced service quality, and diminished convenience in exchange for diffuse, uncertain, and largely invisible gains in collective data autonomy?

This puzzle exposes a microfoundational gap between two otherwise productive literatures. International relations scholarship has illuminated how states instrumentalize digital infrastructure, regulatory extraterritoriality, and platform dependence as instruments of statecraft (Farrell and Newman, 2019; Newman and Posner, 2018; Slaughter, 2017), how the United States, China, and the European Union are reconfiguring chokepoints across cloud, semiconductor, and AI infrastructure (Aaronson, 2023; Bradford, 2023; Farrell and Newman, 2023), and how domestic institutions and sectoral configurations mediate these contests (Bradford et al., 2025; Patil et al., 2025; Repetto, 2025). Yet this work remains predominantly state-centric: it explains why governments pursue data sovereignty agendas but offers limited theoretical purchase on why ordinary citizens, who bear the diffuse costs of such policies, come to endorse them. Information systems and behavioral research, conversely, has produced a sophisticated account of individual-level privacy trade-offs (Dinev and Hart, 2006; Smith et al., 2011; Xu et al., 2011), but has largely modeled the relevant choice as a transaction between an individual and a service provider rather than a judgment about a country’s collective infrastructural posture. Neither tradition, taken alone, can adequately explain support for costly data protection policies in which the locus of risk is geopolitical rather than commercial, and the locus of benefit is collective rather than personal.

This study addresses that gap by theorizing and empirically testing a microfoundational account in which Perceived Foreign Data Dependency (PFDD) functions as a cognitive bridge between macro-level geopolitical conditions and individual-level willingness to accept the costs of data protection. Drawing on the broader literature on costly policy support—including the willingness of citizens to bear material burdens for environmental protection, public health, and national security objectives (Funk, 2000; Hainmueller and Hiscox, 2010; Stadelmann-Steffen and Dermont, 2018)—we argue that data protection policies belong to the same family of valence-laden, collective-good provisions whose support cannot be reduced to immediate personal utility. Specifically, we examine how PFDD shapes two parallel evaluative pathways: a risk-oriented pathway routed through Perceived Cross-border Data Risk (PCDR), and an opportunity-oriented pathway routed through Tolerance for Cross-Border Data Openness (TCDO), both of which in turn shape Costly Data Protection Acceptance (CDPA). To adjudicate between substitute and complement accounts of self-efficacy, we further test Data Self-Efficacy (DSE)—a data-specific operationalisation of the broader digital-capability construct, focusing on individuals’ confidence in handling, interpreting, and controlling their own data—as a moderator of these pathways, asking whether perceived personal capability dampens or amplifies the translation of structural perception into policy preference.

We test this framework using survey data from 620 Korean respondents, analysed via partial least squares structural equation modelling (PLS-SEM). The Republic of Korea offers an analytically informative setting for three reasons. First, with internet penetration above 97% and per-capita data consumption among the highest in the OECD, Korean respondents have substantive lived experience of cross-border digital services on which to ground their judgments (National Information Society Agency, 2024; Organisation for Economic Co-operation and Development, 2024). Second, Korean citizens have been exposed to a sustained public discourse about foreign platform reliance, anchored in concrete and visible episodes such as the long-running dispute over geospatial data export that constrains Google Maps within Korea (Ahn and Ha, 2025), and intensified since 2023 by high-profile PIPC investigations into generative AI providers and successive national initiatives framing dependence on foreign digital infrastructure as a strategic vulnerability (Personal Information Protection Commission, 2024). Third, Korea’s geopolitical position—densely interdependent with both the United States and China while exposed to acute regional security pressures—offers a sharper test of whether geopolitical perception meaningfully shapes individual data-policy preferences than would a country in which such pressures are diffuse or latent.

The contributions of this study are threefold and, in combination, sharpen the puzzle introduced above. First, the paper offers a microfoundational extension of the geopolitics-of-data literature: it demonstrates that the structural conditions identified by Farrell and Newman (2019) operate not only through state-level strategic calculation but also through citizen-level perceptual mediation, and it specifies Perceived Foreign Data Dependency (PFDD) as a measurable construct that connects international relations scholarship with privacy decision research that has rarely engaged it. Second, rather than positing risk and opportunity as competing channels, the paper develops and tests a complementary dual-pathway model in which Perceived Cross-border Data Risk (PCDR) and Tolerance for Cross-Border Data Openness (TCDO) jointly translate PFDD into Costly Data Protection Acceptance—providing one of the first individual-level accounts of why citizens accept the costs of digital sovereignty policies and showing that the two pathways add to, rather than offset, one another. Third, and most distinctively, the paper advances the Enlightenment-Vigilance hypothesis as a reconfiguration of the long-standing substitute-versus-complement debate over digital capability: high-capability citizens do not produce a discriminating profile that partitions legitimate openness benefits from illegitimate dependency risks; instead, capability yields an integrative-vigilance profile in which the same registered dependency triggers a concerted downward revision of the normative legitimacy of cross-border openness itself. This reframing speaks directly to whether digital capability empowers political voice or substitutes for it, and to how governments and platform operators should communicate about cross-border data governance when the relevant audience is not less discerning but more so. The remainder of the paper is organized as follows. Section 2 develops the theoretical framework and hypotheses; Section 3 details the research design and measurement; Section 4 presents the PLS-SEM results; and Section 5 discusses theoretical, policy, and methodological implications.

## 2 Literature review

### 2.1 The geopolitics of data: from statecraft to citizen cognition

International relations scholarship has long treated data and digital infrastructure as objects of statecraft. Building on the tradition of economic interdependence as a source of both vulnerability and leverage, Farrell and Newman (2019) argued that the global digital economy has produced asymmetric chokepoints—central nodes in payments, communications, and data flows—that powerful states can weaponize against rivals and allies alike. Legal scholarship has since recast this dynamic as “data warfare,” in which data itself becomes a contested domain of interstate conflict whose regulatory governance lags the pace of cross-border flows, sharpening the very sovereignty tensions at issue here (Mone et al., 2024). Subsequent work has extended this framework along two complementary lines. The first traces how the United States, China, and the European Union have reorganized cloud, semiconductor, and AI infrastructure into instruments of regulatory and strategic projection (Aaronson, 2023; Bradford, 2023; Farrell and Newman, 2023), while the second locates the formation of national digital policy in the interaction between geopolitical pressure and domestic institutional configuration (Bradford et al., 2025; Newman and Posner, 2018; Slaughter, 2017). Recent conceptual work has further argued that digital sovereignty is best understood as a multi-dimensional condition spanning legal jurisdiction, infrastructural control, and epistemic asymmetry, in which Westphalian territorial authority must be recalibrated for an order organized around private digital infrastructure (Akıllı and Shkurti Özdemir, 2025; Chung, 2026; Santaniello, 2025).

What this scholarship has not provided is a microfoundational account of why the citizens whose welfare is implicated by these policies—through higher prices, slower services, and reduced platform access—come to support them. The implicit assumption in much of this work is that public attitudes either follow elite cues or aggregate national interests through electoral mechanisms, leaving the cognitive process by which individuals translate diffuse geopolitical conditions into concrete policy preferences largely unspecified. Yet if data sovereignty agendas depend for their durability on public acquiescence to non-trivial costs, the legitimacy of these agendas rests on a perceptual mechanism that the field has yet to theorize. This study locates that mechanism in citizens’ subjective construal of foreign data dependency: the cognitive registration of the country’s reliance on externally controlled platforms, infrastructures, and AI systems, which we develop in §2.3 as Perceived Foreign Data Dependency (PFDD). Before introducing PFDD, however, we first examine what the dominant individual-level account of data-related decision-making—privacy calculus—can and cannot tell us about support for costly data protection.

### 2.2 Privacy calculus and its limits in the geopolitical era

Within information systems and behavioral research, privacy calculus has provided the dominant framework for analysing individual data-related decisions for nearly two decades. In its canonical form, the framework holds that individuals weigh the perceived risks of disclosure against the anticipated benefits of participation, and disclose or withhold accordingly (Dinev and Hart, 2006). The Antecedents-Privacy Concerns-Outcomes (APCO) macro-model further organized this logic by specifying how dispositional, environmental, and informational antecedents shape privacy concerns, which in turn shape behavioral outcomes (Smith et al., 2011), with subsequent refinements emphasizing the role of context and the situational construction of privacy concerns (Xu et al., 2011). Two persistent empirical patterns have shaped how this literature has evolved: the privacy paradox—the gap between stated concern and observed disclosure—has invited a sustained debate over whether the calculus is genuinely calculative, heuristic, or normatively constrained (Acquisti et al., 2015; Kokolakis, 2017).

A growing wave of post-2024 scholarship has extended privacy calculus into the generative AI context, finding that disclosure intentions on AI-generated content platforms remain shaped by familiar calculus variables, with users continuing to weigh perceived risks against personalization benefits when interacting with chatbots (Zhou and Tang, 2025), that awareness of AI-specific privacy threats remains uneven among ChatGPT users (Dahabiyeh et al., 2026), and that, on social media, heightened privacy concern weakens rather than strengthens the translation of disclosure awareness into protective behavior, a pattern the authors read as disengagement or privacy fatigue (Mazhar et al., 2026). These extensions demonstrate the framework’s continued generativity, but they preserve a structural feature that limits its reach for our purposes: the relevant choice is consistently modeled as a transaction between an individual and a service provider. The risks are personal—identity exposure, secondary use, profiling—and the benefits are personal—convenience, personalization, social participation. The framework offers no purchase on a different class of judgments, in which the relevant risk is geopolitical rather than commercial, the relevant benefit is collective rather than individual, and the relevant action is policy support rather than disclosure. To explain why citizens accept costly data protection policies whose benefits accrue to national infrastructural autonomy, the calculus must be coupled to a perceptual antecedent that registers structural rather than transactional dependency. We turn to that antecedent next.

### 2.3 Perceived foreign data dependency (PFDD) as a cognitive bridge

We define Perceived Foreign Data Dependency (PFDD) as an individual’s subjective assessment of the degree to which their country relies on externally controlled platforms, infrastructures, and AI systems for the storage, transmission, and processing of personal and societal data. PFDD is a perceptual rather than objective construct: two citizens of the same country, exposed to the same infrastructural reality, may register dependency very differently depending on their attention to public discourse, their concrete experiences with cross-border services, and their broader political dispositions. The construct is informed by, but distinct from, three related notions in the literature. First, it is anchored in but more specific than generalized techno-nationalism or anti-foreign sentiment, in that it concerns the cognitive registration of a particular structural condition—data and infrastructural reliance—rather than affective orientation toward foreign actors as such. Second, it differs from platform-level distrust in that its referent is the country’s aggregate posture rather than the trustworthiness of any individual provider. Third, it differs from perceived vulnerability in privacy calculus in that it concerns collective rather than personal exposure.

Recent conceptual work on digital sovereignty provides direct theoretical traction for treating PFDD as a meaningful object of measurement. Chung (2026) identifies infrastructural dependence on dominant platforms and epistemic asymmetry vis-à-vis foreign-controlled AI systems as the two principal mechanisms through which contemporary sovereignty deficits are produced—mechanisms that, by their nature, are visible to citizens through everyday digital experience. Sectoral and governance studies further demonstrate that dependency manifests across distinct domains, including financial systems and data sovereignty regulation (Patil et al., 2025), and cross-border regulatory architectures whose conceptual boundaries remain actively contested (Repetto, 2025). PFDD captures, at the individual cognitive level, what these structural analyses describe at the systemic level: a registration that critical data-related capabilities are not under domestic control. As a cognitive bridge, PFDD is positioned to translate macro-level geopolitical conditions into micro-level evaluative responses—but it does so through specific evaluative pathways, not through a single undifferentiated effect. We elaborate these pathways in the next section.

### 2.4 Dual pathways: risk (PCDR) and opportunity (TCDO)

We argue that PFDD shapes Costly Data Protection Acceptance (CDPA) through two parallel evaluative pathways: a risk-oriented pathway routed through Perceived Cross-border Data Risk (PCDR), and an opportunity-oriented pathway routed through Tolerance for Cross-Border Data Openness (TCDO). The dual-pathway logic draws on a long tradition of dual-process accounts of policy attitude formation, in which the same structural condition can be appraised through threat-related and benefit-related construals that operate in parallel rather than as competing reductions of one another. We treat CDPA as the dependent construct: an individual’s stated willingness to accept tangible personal costs—higher prices, reduced service quality, diminished convenience—in exchange for stronger national or regional data protection. CDPA belongs to the broader family of costly policy support, alongside willingness to bear material burdens for environmental protection, public health, and national security objectives, and it inherits the analytic insight that support for collective-good provisions cannot be reduced to immediate personal utility (Funk, 2000; Hainmueller and Hiscox, 2010; Stadelmann-Steffen and Dermont, 2018).

The risk-oriented pathway rests on a straightforward inference. When citizens register that critical data-related capabilities lie under foreign control, they have grounds to perceive cross-border data flows as exposing them—and the collective—to risks they cannot directly govern: surveillance by foreign states, secondary use by foreign firms, and data weaponization in geopolitical disputes. PCDR captures this risk-oriented appraisal of cross-border data flows. Higher PFDD therefore raises PCDR (H1), and the heightened PCDR in turn raises CDPA, because stronger protection becomes a means of insulating the collective from a recognized source of risk (H3). We accordingly hypothesize:

*H1*: Perceived Foreign Data Dependency has a positive effect on Perceived Cross-Border Data Risk.

*H3*: Perceived Cross-Border Data Risk has a positive effect on Costly Data Protection Acceptance.

The opportunity-oriented pathway operates with the opposite valence. TCDO captures an individual’s willingness to accept—and tolerate the trade-offs entailed by—the continued openness of cross-border data flows. Citizens who hold a high tolerance for cross-border openness place priority on the productivity, innovation, and welfare benefits of free data movement and are inclined to live with its attendant exposure rather than constrain it. As PFDD rises, however, the structural exposure that openness implies becomes more salient: registering that the country’s data-related capabilities sit under foreign control reduces the willingness to live with that openness, and TCDO declines (H2). Tolerance for openness, in turn, works against costly protection: those who remain comfortable with the openness of cross-border flows have weaker grounds to bear personal costs in pursuit of stronger domestic safeguards, and CDPA is correspondingly lower (H4). Together, these two effects predict that PFDD reduces CDPA indirectly through the suppression of TCDO, even as it raises CDPA indirectly through the elevation of PCDR. We hypothesize:

*H2*: Perceived Foreign Data Dependency has a negative effect on Tolerance for Cross-Border Data Openness.

*H4*: Tolerance for Cross-Border Data Openness has a negative effect on Costly Data Protection Acceptance.

Because both pathways link PFDD to CDPA through distinct evaluative construals, we further specify their mediating role explicitly:

*H5*: Perceived Cross-Border Data Risk mediates the relationship between Perceived Foreign Data Dependency and Costly Data Protection Acceptance.

*H6*: Tolerance for Cross-Border Data Openness mediates the relationship between Perceived Foreign Data Dependency and Costly Data Protection Acceptance.

### 2.5 Data self-efficacy as a moderator: substitute or complement?

Whether the dual pathways translate uniformly across the population is itself a theoretical question. Data Self-Efficacy (DSE)—an individual’s confidence in their own capability to use digital technologies effectively, navigate online environments, and manage data-related risks—is among the most consequential individual-level moderators in this domain. Two opposing accounts of its political effect coexist in the literature. The substitute account, associated with second- and third-level digital divide research, holds that highly capable individuals perceive themselves as able to manage digital risks privately and therefore feel less need for collective protection; capability substitutes for policy support (Hargittai, 2010). The complement account, drawing on civic-engagement traditions in digital citizenship, holds the opposite: capable individuals are more attentive to systemic conditions, more cognitively engaged with policy debate, and more efficacious in supporting collective protective action; capability complements policy support (Park, 2013; van Deursen and Helsper, 2018).

We argue that the substitute and complement accounts have been over-stated as a single, undifferentiated contest, and that the more theoretically productive question is how DSE conditions each of the two construals separately. On the risk-oriented pathway, we expect DSE to operate as a complement: high-DSE citizens, by virtue of greater attentiveness to systemic conditions and more developed cognitive engagement with platform infrastructure, more readily translate registered foreign data dependency into a heightened appraisal of cross-border risk. DSE thus strengthens the front-end translation of PFDD into PCDR. On the openness pathway, however, DSE operates differently. Citizens with greater Data Self-Efficacy possess the cognitive resources to recognize the productivity, innovation, and welfare benefits that openness in cross-border data flows confers, and to distinguish those benefits from the risks generated by dependency. As a result, when PFDD rises, high-DSE citizens are less inclined than their lower-DSE counterparts to withdraw their tolerance for openness; the negative pathway from PFDD to TCDO is correspondingly weakened. Taken together, the two moderation effects predict a complementary cognitive profile in which capable citizens become more risk-aware in the face of dependency without ceasing to value openness.

*H7*: Data Self-Efficacy positively moderates the relationship between Perceived Foreign Data Dependency and Perceived Cross-Border Data Risk, such that the effect of PFDD on PCDR is stronger at higher levels of DSE.

*H8*: Data Self-Efficacy negatively moderates the relationship between Perceived Foreign Data Dependency and Tolerance for Cross-Border Data Openness, such that the negative effect of PFDD on TCDO is weaker at higher levels of DSE.

Taken together, H1–H4 specify the dual-pathway translation of PFDD into CDPA through PCDR (positive) and TCDO (negative); H5–H6 formalize the mediating role of these two construals; and H7–H8 specify how DSE conditions the front-end translation of PFDD into each evaluative construal, with directional predictions that together describe a complementary high-DSE profile of heightened risk-awareness alongside sustained tolerance for openness. Figure 1 summarizes the resulting research framework, in which PFDD operates as the cognitive bridge between macro-level geopolitical conditions and micro-level policy preference, PCDR and TCDO operate as parallel mediating evaluative construals, and DSE operates as a moderator on the PFDD-to-construal pathways. The remainder of the paper tests this framework using survey data from Korean respondents.

**Figure 1:**
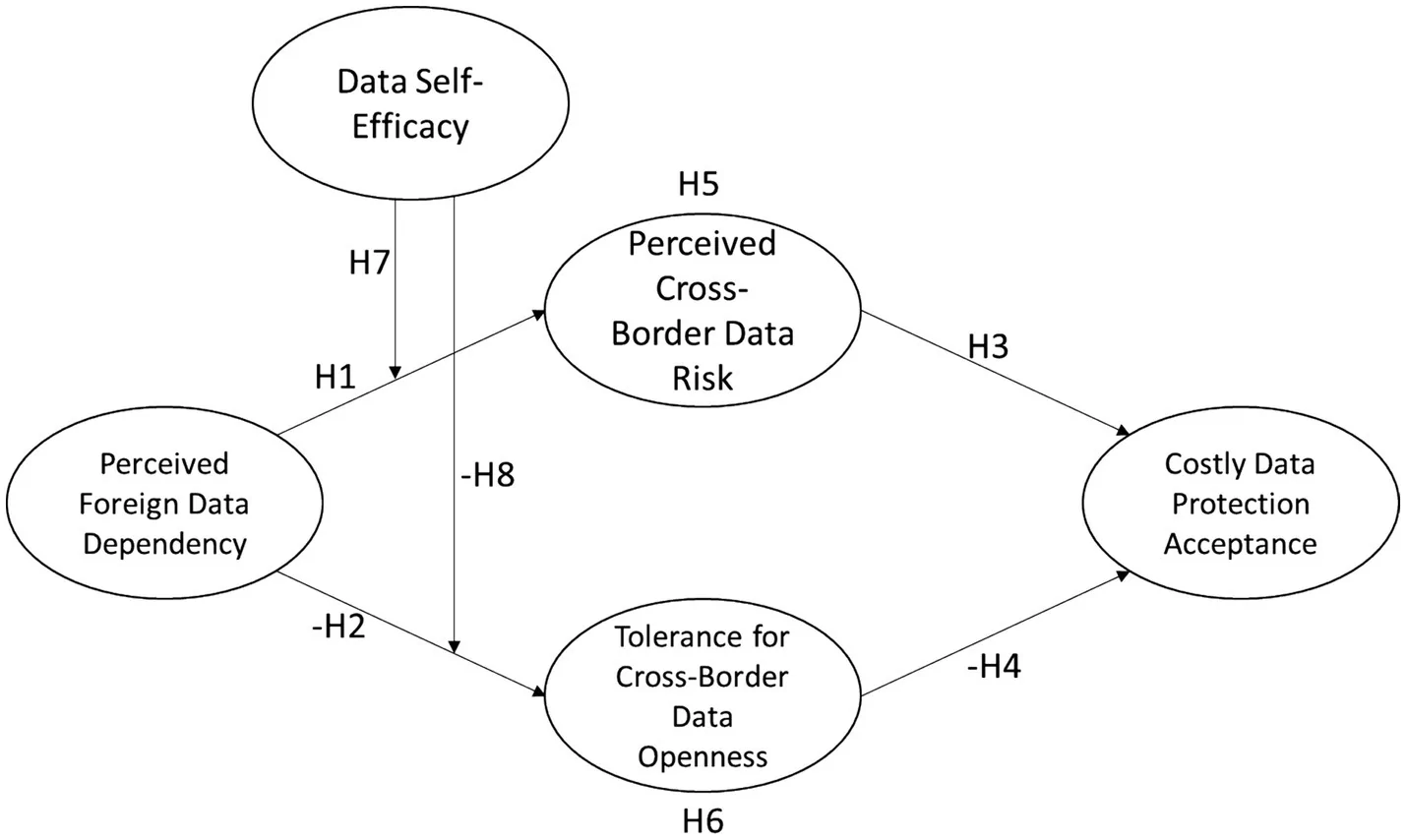
Research model.

## 3 Methodology

### 3.1 Research subjects and data collection

This study targets the adult population residing in South Korea as the unit of analysis, as individuals represent the primary stakeholders in cross-border data flows and are directly affected by data governance policies in contemporary digital environments. Given the study’s focus on individuals’ cognitive evaluations, attitudinal orientations, and behavioral acceptance, a survey-based quantitative approach was adopted. Data were collected through a professional online survey platform, Embrain^1^, which maintains a large and diverse panel of respondents in South Korea and supports advanced sampling and quality control functions suitable for academic research. A targeted (purposive) sampling strategy combined with quota sampling was employed to ensure balanced representation across key demographic dimensions, including gender, age, and residential region (capital vs. non-capital areas), based on the general population structure. To ensure respondent eligibility, participants were required to be aged 18–59 and currently residing in South Korea, and screening procedures were implemented to automatically exclude ineligible respondents. To enhance data quality, multiple procedural controls were applied, including restrictions on duplicate responses through IP and device monitoring, the inclusion of attention-check items to identify inattentive responses, and the use of minimum response time thresholds to filter out invalid questionnaires.

In addition, respondents who completed the survey were provided with appropriate monetary incentives through the platform, which has been shown to improve response rates and data reliability in online panel research. Considering the structural complexity of the proposed model, which involves multiple latent constructs as well as mediation and moderation effects, an adequate sample size was required to ensure statistical power and model stability. Following established guidelines for structural equation modeling, the minimum sample size was determined based on the ratio of measurement items to observations. With 25 measurement items included in the questionnaire, the target sample size was set between 350 and 450 valid responses to ensure robust parameter estimation and reliable hypothesis testing. After data screening and the removal of invalid responses, a total of 620 valid questionnaires were retained for subsequent analysis, substantially exceeding the minimum threshold and providing sufficient statistical power for the structural equation modeling procedures employed in this study.

### 3.2 Measurement

This study adopts a structured measurement design that integrates both demographic variables and latent constructs to capture respondents’ socio-demographic characteristics as well as their perceptions, attitudes, and behavioral tendencies toward cross-border data governance. The measurement framework is grounded in the distinction between observable background attributes and theoretically derived latent variables, thereby ensuring both descriptive completeness and analytical rigor.

#### 3.2.1 Demographic variables

Demographic variables were included to reflect key dimensions of population structure and to align with the quota sampling design adopted in this study. These variables serve a descriptive and contextual role, enabling the characterization of the sample and facilitating group-based comparisons where relevant. In contrast, the focal constructs were conceptualized as latent variables representing underlying cognitive evaluations, attitudinal orientations, and behavioral acceptance. These constructs were operationalized using multi-item reflective measures to capture abstract theoretical concepts through observable indicators. Specifically, the questionnaire collected information on respondents’ gender, age, residential region (capital vs. non-capital areas), and educational attainment. These variables represent fundamental dimensions of socio-demographic structure and are closely associated with variations in digital access and exposure. While these demographic variables are not part of the theoretical model, they provide an important contextual basis for interpreting the empirical results.

#### 3.2.2 Research variables

All latent constructs in this study were measured using multi-item reflective scales on a seven-point Likert scale ranging from 1 (“strongly disagree”) to 7 (“strongly agree”). To maintain conceptual coherence across constructs, all items were consistently anchored in the context of cross-border data flows, thereby aligning cognitive, attitudinal, and behavioral dimensions within a unified analytical framework. The measurement approach combined the adaptation of established scales with theory-driven item development. Constructs with well-established measurement traditions, such as perceived risk and self-efficacy, were adapted from validated instruments and contextualized to cross-border data environments. In contrast, constructs that are more specific to the present research context—namely perceived foreign data dependency, tolerance for cross-border data openness, and costly data protection acceptance—were operationalized through items developed directly from their theoretical definitions, ensuring content validity and conceptual precision.

Prior to fielding, the instrument underwent a structured development and validation process. For the three constructs developed specifically for this study—perceived foreign data dependency, tolerance for cross-border data openness, and costly data protection acceptance—an initial item pool was generated directly from the constructs’ theoretical definitions and then reviewed by a panel of subject-matter experts, whose assessments of item relevance and clarity were used to refine wording and establish content validity. The refined instrument was subsequently administered in a pretest, and items were revised on the basis of the results before the main survey. The questionnaire was originally developed in Chinese by the research team and administered in Korean, since the target population comprised South Korean adults; a forward- and back-translation procedure was employed, in which an independent bilingual translator back-translated the Korean version and discrepancies with the original were reconciled prior to fielding. The complete item set, translated into English, is provided in the Supplementary Material. The psychometric adequacy of all scales—including the newly developed constructs—was then formally assessed on the main sample through the internal-consistency reliability, convergent-validity, and discriminant-validity procedures reported in Sections 4.2 and 4.3.

(1) Perceived Foreign Data Dependency

Perceived foreign data dependency captures individuals’ cognitive assessment of the extent to which domestic data infrastructures rely on foreign-controlled systems, including data storage, processing, and platform operations. This construct is conceptually grounded in the notion of structural dependence in global networked systems (Farrell and Newman, 2019), but is operationalized at the level of individual perception.

(2) Perceived Cross-Border Data Risk

Perceived cross-border data risk reflects individuals’ subjective evaluation of uncertainties and potential harms associated with the cross-border movement and storage of data. The measurement was adapted from established privacy concern scales (Dinev and Hart, 2004, 2006), with items modified to reflect risks arising from jurisdictional complexity, loss of control, and accountability challenges.

(3) Tolerance for Cross-Border Data Openness

Tolerance for cross-border data openness represents the extent to which individuals are willing to accept ongoing cross-border data flows and the presence of foreign data infrastructures despite perceived risks. This construct is informed by the literature on tolerance and openness under perceived threat (Hainmueller and Hiscox, 2006, 2010), and is operationalized as an attitudinal disposition distinct from policy support.

(4) Costly Data Protection Acceptance

Costly data protection acceptance captures individuals’ willingness to endorse restrictive data governance measures under explicit personal cost conditions. This construct is conceptually grounded in the literature on policy trade-offs and willingness to bear personal costs for collective or long-term benefits (Funk, 2000; Stadelmann-Steffen and Dermont, 2018). Prior research suggests that individuals’ support for public policies often depends on their readiness to accept associated personal sacrifices, such as financial costs, reduced convenience, or constraints on individual freedom. Drawing on this logic, the measurement design explicitly incorporates cost elements—such as reduced convenience, increased financial burden, and potential disruption to daily activities—thereby distinguishing substantive acceptance from symbolic agreement.

(5) Data Self-Efficacy

Finally, Data Self-Efficacy reflects individuals’ perceived capability to manage and protect their personal data in digital environments. This construct was adapted from the Internet self-efficacy framework (Eastin and LaRose, 2000), with emphasis placed on perceived control, competence, and confidence in data-related decision-making.

### 3.3 Data analysis

This study employed partial least squares structural equation modeling (PLS-SEM) to test the proposed research model and hypotheses. PLS-SEM was selected as the primary analytical approach due to its suitability for analyzing complex models that involve multiple latent constructs, as well as mediation and moderation effects. In addition, PLS-SEM is particularly appropriate for prediction-oriented research and does not impose strict distributional assumptions on the data (Hair et al., 2021). The data analysis procedure followed a two-step approach, consisting of the assessment of the measurement model and the structural model. First, the measurement model was evaluated to ensure reliability and validity. Internal consistency reliability was assessed using Cronbach’s alpha and composite reliability (CR), with values exceeding the recommended threshold of 0.70 indicating satisfactory reliability. Convergent validity was examined using average variance extracted (AVE), with values above 0.50 indicating adequate convergence of the indicators.

Discriminant validity was assessed using the heterotrait–monotrait ratio (HTMT), with values below 0.85 used as the threshold for establishing discriminant validity (Henseler et al., 2015). Second, the structural model was evaluated to examine the hypothesized relationships among constructs. Path coefficients, *t*-values, and *p*-values were obtained using a bootstrapping procedure with 5,000 resamples, as recommended in prior research (Hair et al., 2021). The significance of direct effects (H1–H4), mediation effects (H5–H6), and moderation effects (H7–H8) was assessed based on the bootstrapped confidence intervals.

Mediation effects were tested by examining the indirect effects of perceived foreign data dependency on costly data protection acceptance through perceived cross-border data risk and tolerance for cross-border data openness. A mediation effect was considered significant when the bootstrapped confidence interval did not include zero. Moderation effects were tested by creating interaction terms between Data Self-Efficacy and the independent variable, following the product indicator approach commonly used in PLS-SEM. To assess overall model quality, explanatory power was evaluated using the coefficient of determination (*R*^2^) for the endogenous constructs, together with the goodness-of-fit (GoF) index. Collinearity in the structural model was assessed using the variance inflation factor (VIF), with values below 3.3 indicating that collinearity is unlikely to pose a serious concern in this study.

## 4 Results

### 4.1 Sample profile and demographic characteristics

As reported in Table 1, the sample is approximately balanced across gender and broadly distributed across the four major adult age bands, with middle-aged respondents forming a slight plurality. Residence skews toward non-capital regions, reducing the metropolitan concentration that often characterizes online panel surveys in South Korea, while educational attainment is high, with the large majority holding at least a bachelor’s degree. The composition supports analytical generalisability across gender, age cohort, and region, with the caveat that the educated profile should be borne in mind when interpreting effects in which digital capability plays a role.

**Table 1:** Demographic profile of respondents (*N* = 620).

Demographic variable	Category	Frequency (*n*)	Percentage (%)
Gender	Male	303	48.9
	Female	317	51.1
Age group	18–29	141	22.7
	30–39	150	24.2
	40–49	154	24.8
	50–59	175	28.2
Area of residence	Capital region	190	30.6
	Non-capital region	430	69.4
Education level	High school or below	30	4.8
	Junior college	85	13.7
	Bachelor’s degree	264	42.6
	Graduate school or above	241	38.9

### 4.2 Measurement model assessment: reliability and convergent validity analysis

Table 2 reports the measurement model assessment. Across all five constructs, outer loadings comfortably exceed the 0.708 benchmark recommended by Hair et al. (2022), so no item is dropped. Cronbach’s alpha and composite reliability both sit well above the conventional 0.70 cut-off (Nunnally and Bernstein, 1994; Hair et al., 2022), indicating strong internal consistency, and the average variance extracted (AVE) values clear the 0.50 Fornell and Larcker (1981) threshold for every construct. Indicator reliability, internal consistency, and convergent validity are therefore established for each of the five latent constructs, providing a sound measurement foundation for the structural analysis that follows.

**Table 2:** Measurement model assessment: Reliability and convergent validity analysis.

Construct	Item	Outer loadings	Cronbach’s alpha	Composite reliability	AVE
Costly Data Protection Acceptance (CDPA)	CDPA1	0.841	0.911	0.933	0.735
	CDPA2	0.857			
	CDPA3	0.869			
	CDPA4	0.867			
	CDPA5	0.850			
Data Self-Efficacy (DSE)	DSE1	0.825	0.914	0.934	0.740
	DSE2	0.863			
	DSE3	0.875			
	DSE4	0.873			
	DSE5	0.862			
Perceived Cross-Border Data Risk (PCDR)	PCDR1	0.837	0.916	0.936	0.745
	PCDR2	0.862			
	PCDR3	0.857			
	PCDR4	0.880			
	PCDR5	0.880			
Perceived Foreign Data Dependency (PFDD)	PFDD1	0.827	0.915	0.935	0.744
	PFDD2	0.865			
	PFDD3	0.863			
	PFDD4	0.876			
	PFDD5	0.879			
Tolerance for Cross-Border Data Openness (TCDO)	TCDO1	0.799	0.903	0.927	0.717
	TCDO2	0.864			
	TCDO3	0.855			
	TCDO4	0.852			
	TCDO5	0.863			

### 4.3 Discriminant validity assessment (HTMT)

As shown in Table 3, all pairwise HTMT ratios fall well below the strict 0.85 and the more permissive 0.90 thresholds recommended by Henseler et al. (2015), with the highest ratio observed for the PCDR–CDPA pair. The five latent constructs—CDPA, DSE, PCDR, PFDD, and TCDO—are therefore empirically distinct and free of problematic conceptual overlap, satisfying discriminant validity requirements for the structural model.

**Table 3:** Discriminant validity assessment (HTMT).

Construct	CDPA	DSE	PCDR	PFDD	TCDO
CDPA					
DSE	0.470				
PCDR	0.551	0.447			
PFDD	0.529	0.520	0.493		
TCDO	0.489	0.473	0.522	0.523	

CDPA, Costly Data Protection Acceptance; DSE, Data Self-Efficacy; PCDR, Perceived Cross-Border Data Risk; PFDD, Perceived Foreign Data Dependency; TCDO, Tolerance for Cross-Border Data Openness.

### 4.4 Goodness-of-fit assessment

The Goodness of Fit (GoF) index provides a global criterion for the joint performance of the measurement and structural models, defined as the geometric mean of average communality (AVE) and average *R*^2^. The index follows the formula GoF = √(AVE × *R*^2^). Esposito Vinzi et al (2010) propose thresholds of 0.10, 0.25, and 0.36 for weak, medium, and strong fit. The computed GoF, reported below, comfortably surpasses the strong-fit benchmark, indicating that the integrated model accounts for a substantial share of variance across the constructs.


$$\mathrm{GoF}=\surd (\mathrm{AVE}\times R^{2})=\surd (0.736\times 0.383)=0.531$$

### 4.5 Structural model assessment and hypothesis testing

As reported in Table 4, every hypothesized structural path is statistically significant at *p* < 0.001, with *T*-values comfortably exceeding the recommended threshold of 1.96 (Hair et al., 2022). Perceived Foreign Data Dependency raises Perceived Cross-Border Data Risk and depresses Tolerance for Cross-Border Data Openness, and the interaction with Data Self-Efficacy strengthens both effects in the same direction. The two evaluative channels then translate into Costly Data Protection Acceptance with opposite signs—risk perception increasing acceptance, openness tolerance reducing it. Explained variance for the three endogenous constructs falls in the 0.35–0.41 range, exceeding the moderate-explanatory benchmark proposed by Chin (1998) and indicating that the dual-pathway structure carries substantive predictive weight rather than residual statistical noise (Figures 2–4).

**Table 4:** Structural model assessment and hypothesis testing.

Path	Original sample	Standard deviation	T statistics	*p* values	*R* ^2^
Perceived Foreign Data Dependency → Perceived Cross-Border Data Risk	0.373	0.042	8.971	<0.001	0.389
Data Self-Efficacy × Perceived Foreign Data Dependency → Perceived Cross-Border Data Risk	0.299	0.029	10.342	<0.001	
Data Self-Efficacy × Perceived Foreign Data Dependency → Tolerance for Cross-Border Data Openness	−0.273	0.031	8.670	<0.001	0.407
Perceived Foreign Data Dependency → Tolerance for Cross-Border Data Openness	−0.393	0.038	10.244	<0.001	
Perceived Cross-Border Data Risk → Costly Data Protection Acceptance	0.387	0.036	10.703	<0.001	0.353
Tolerance for Cross-Border Data Openness → Costly Data Protection Acceptance	−0.292	0.037	7.810	<0.001	

R² refers to the dependent construct of each structural block.

**Figure 2:**
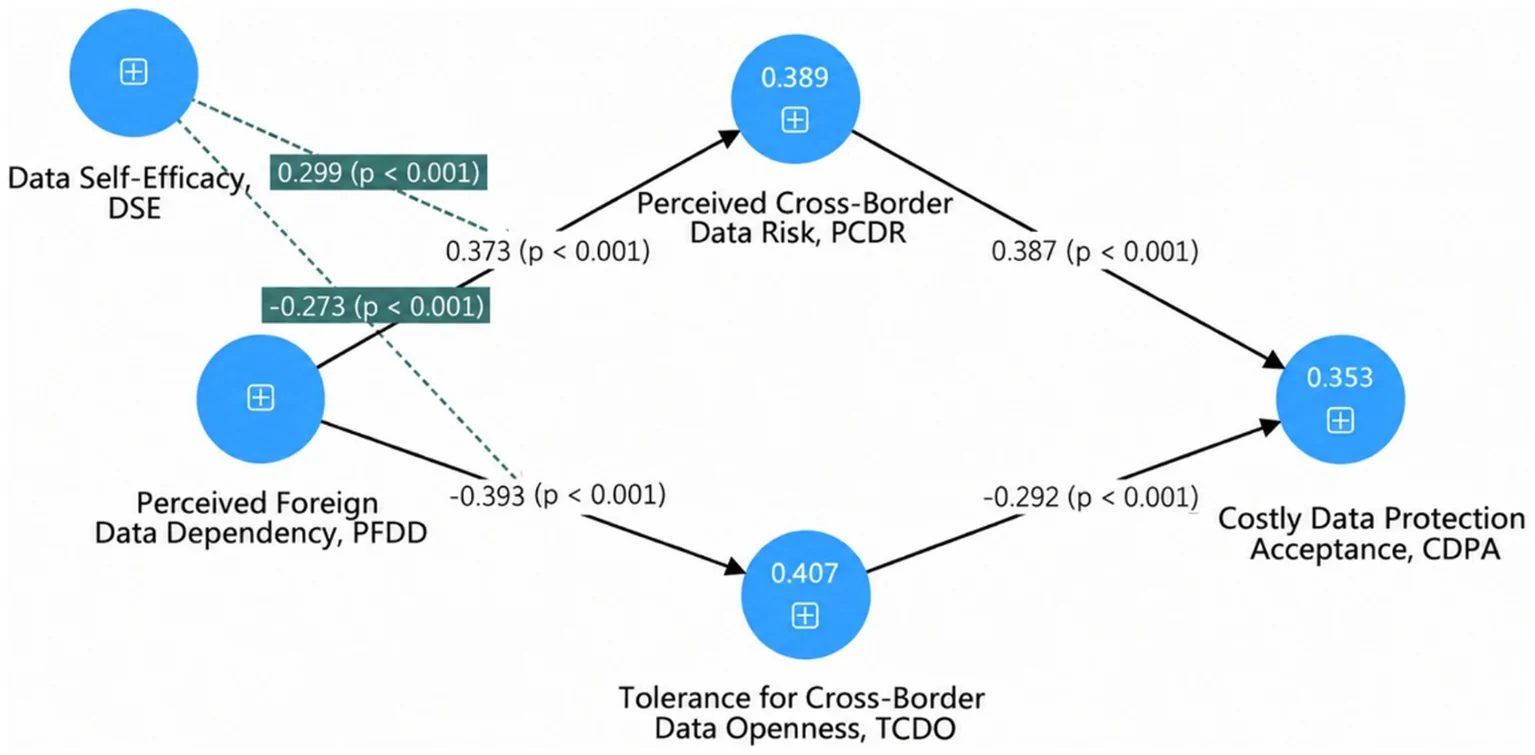
Structural model estimation results: Path coefficients and hypothesis testing.

**Figure 3:**
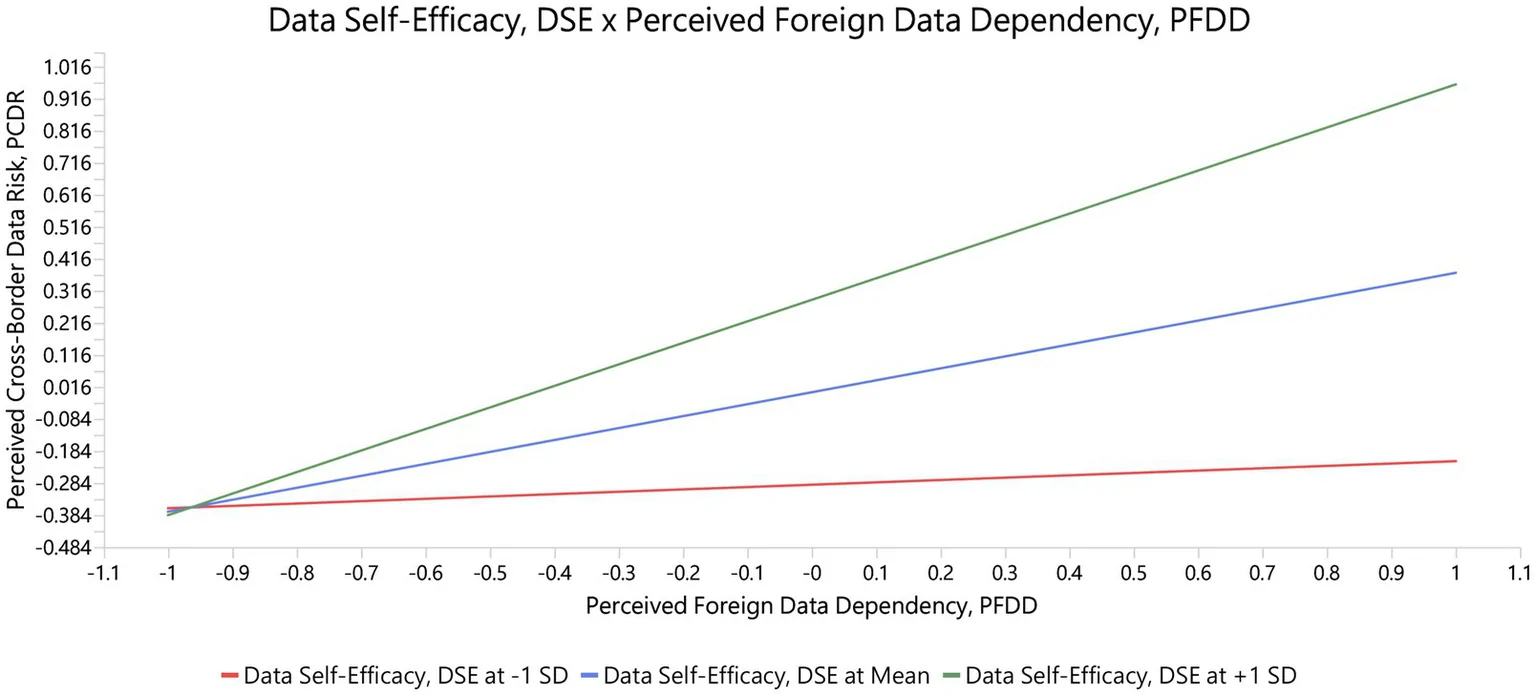
Moderating effect of Data Self-Efficacy on the relationship between perceived foreign data dependency and perceived cross-border data risk.

**Figure 4:**
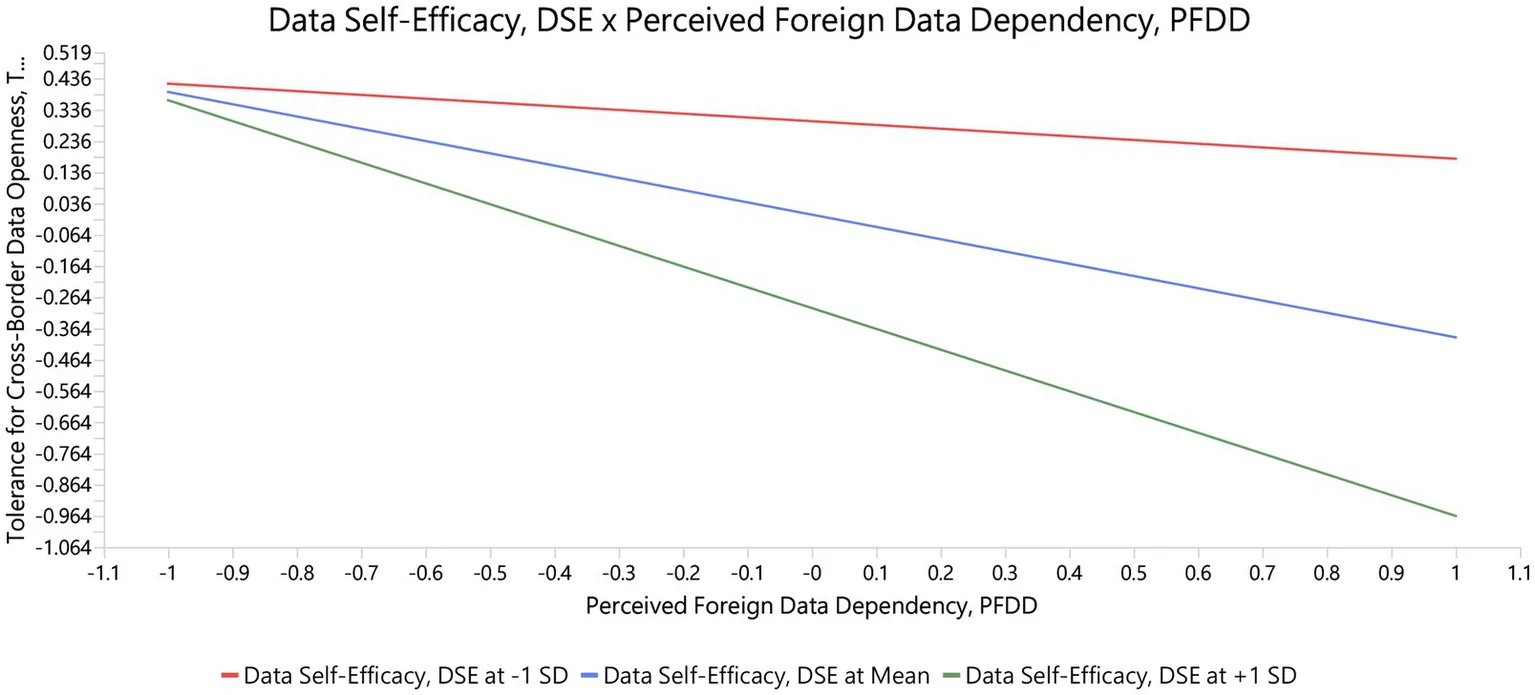
Moderating effect of Data Self-Efficacy on the relationship between perceived foreign data dependency and tolerance for cross-border data openness.

### 4.6 Effect size assessment (*f*^2^)

Effect-size assessment using Cohen's (1988) benchmarks (0.02 small, 0.15 medium, 0.35 large) places every *f*^2^ values in the small-to-medium range, with three paths reaching the medium benchmark, as detailed in Table 5. The strongest contributions come from Perceived Foreign Data Dependency to both mediators and from Perceived Cross-Border Data Risk to Costly Data Protection Acceptance, identifying these as the model’s primary substantive drivers. The interaction terms involving Data Self-Efficacy register smaller but non-trivial *f*^2^ values, consistent with their role as second-order moderators rather than principal predictors. Collectively, the pattern confirms that the dual-pathway structure carries practical, not merely statistical, relevance.

**Table 5:** Effect size assessment (*f*^2^).

Path	*f* ^2^
Data Self-Efficacy → Perceived Cross-Border Data Risk	0.099
Data Self-Efficacy → Tolerance for Cross-Border Data Openness	0.109
Data Self-Efficacy × Perceived Foreign Data Dependency → Perceived Cross-Border Data Risk	0.138
Data Self-Efficacy × Perceived Foreign Data Dependency → Tolerance for Cross-Border Data Openness	0.118
Perceived Cross-Border Data Risk → Costly Data Protection Acceptance	0.168
Perceived Foreign Data Dependency → Perceived Cross-Border Data Risk	0.164
Perceived Foreign Data Dependency → Tolerance for Cross-Border Data Openness	0.188
Tolerance for Cross-Border Data Openness → Costly Data Protection Acceptance	0.095

### 4.7 Collinearity assessment (VIF)

Collinearity diagnostics, summarized in Table 6, return Variance Inflation Factor values for every predictor and interaction term well below both the conservative threshold of 3.3 (Kock, 2015) and the standard ceiling of 5.0 (Hair et al., 2022). Multicollinearity therefore poses no concern for the structural model, and the path coefficients reported above can be interpreted as stable, independent contributions of each construct.

**Table 6:** Collinearity assessment (VIF).

Path	VIF
Data Self-Efficacy → Perceived Cross-Border Data Risk	1.379
Data Self-Efficacy → Tolerance for Cross-Border Data Openness	1.379
Data Self-Efficacy × Perceived Foreign Data Dependency → Perceived Cross-Border Data Risk	1.009
Data Self-Efficacy × Perceived Foreign Data Dependency → Tolerance for Cross-Border Data Openness	1.009
Perceived Cross-Border Data Risk → Costly Data Protection Acceptance	1.378
Perceived Foreign Data Dependency → Perceived Cross-Border Data Risk	1.383
Perceived Foreign Data Dependency → Tolerance for Cross-Border Data Openness	1.383
Tolerance for Cross-Border Data Openness → Costly Data Protection Acceptance	1.378

### 4.8 Mediation effect assessment

Bootstrap-based mediation analysis confirms that Perceived Foreign Data Dependency transmits its influence on Costly Data Protection Acceptance through both mediating channels, with bias-corrected 95% confidence intervals excluding zero in each case (Hair et al., 2022; Zhao et al., 2010). The risk-oriented indirect path operates by elevating Perceived Cross-Border Data Risk, while the opportunity-oriented indirect path operates by suppressing Tolerance for Cross-Border Data Openness; the magnitudes and confidence intervals are reported in Table 7. Crucially, both indirect effects share the same sign on the outcome, producing a complementary—rather than competing—dual-mediation structure in which structural perception is translated into costly policy support through two simultaneously activated cognitive channels.

**Table 7:** Mediation effect assessment.

Indirect path	Original sample	Standard deviation	*T* statistics	*p* values	2.50%	97.50%
Perceived Foreign Data Dependency → Perceived Cross-Border Data Risk → Costly Data Protection Acceptance	0.144	0.021	6.782	<0.001	0.106	0.189
Perceived Foreign Data Dependency → Tolerance for Cross-Border Data Openness → Costly Data Protection Acceptance	0.115	0.020	5.719	<0.001	0.078	0.156

## 5 Conclusion and discussion

### 5.1 Summary of findings

This study set out to address a microfoundational puzzle: in an era in which data has become an instrument of geopolitical contestation, why do citizens express willingness to bear material personal costs for collective data protection? Drawing on survey data from 620 Korean respondents and analyzing the data via PLS-SEM, we tested an integrated framework in which Perceived Foreign Data Dependency (PFDD) operates as a cognitive bridge linking macro-level geopolitical conditions to micro-level policy preference through two parallel evaluative construals, with Data Self-Efficacy (DSE) functioning as a competing-hypothesis arbiter on the front-end translation. Seven of the eight hypotheses received empirical support in their predicted direction, and the remaining hypothesis exhibited a statistically significant effect in the opposite direction—a result that, as we elaborate below, sharpens rather than weakens the theoretical contribution.

First, the dual-pathway specification is robustly supported. PFDD significantly increases Perceived Cross-Border Data Risk (*β* = 0.373, *p* < 0.001; H1) and significantly decreases Tolerance for Cross-Border Data Openness (*β* = −0.393, *p* < 0.001; H2). PCDR in turn raises Costly Data Protection Acceptance (*β* = 0.387, *p* < 0.001; H3), while TCDO suppresses it (*β* = −0.292, *p* < 0.001; H4). Both mediating effects (H5, H6) are statistically significant with bootstrap confidence intervals excluding zero (indirect effect through PCDR: *β* = 0.144, 95% CI [0.106, 0.189]; through TCDO: *β* = 0.115, 95% CI [0.078, 0.156]), indicating that PFDD shapes CDPA through complementary—rather than competing—risk and opportunity construals. Together, the structural model explains a non-trivial share of variance in the focal outcome (R^2^ for CDPA = 0.353), and in both mediating construals (R^2^ for PCDR = 0.389; R^2^ for TCDO = 0.407), exceeding the moderate-explanatory benchmark of 0.33 recommended in PLS-SEM practice (Chin, 1998).

Second, the moderation results reveal a theoretically more interesting pattern than the original two-account framing anticipated. On the risk-oriented pathway, DSE strengthens the translation of PFDD into PCDR (*β* = 0.299, *p* < 0.001; H7 supported), consistent with the complement account: digitally capable citizens are more—not less—likely to register foreign dependency as cross-border risk. On the opportunity-oriented pathway, however, DSE does not attenuate the negative effect of PFDD on TCDO as H8 had predicted; instead, it intensifies it (*β* = −0.273, *p* < 0.001), strengthening the already-negative pathway. H8 is therefore not supported in its hypothesized direction, but the interaction term is statistically significant and points in the opposite direction. Far from confirming a ‘complement-on-risk, substitute-on-openness’ profile in which capable citizens retain their tolerance for openness, the data reveal a unified vigilance profile: as foreign dependency becomes salient, digitally capable Koreans both amplify their risk appraisal and withdraw their tolerance for openness with greater force than less capable respondents do. We label this pattern an Enlightenment-Vigilance configuration and develop its theoretical implications in §5.2.

### 5.2 Theoretical contributions

The study advances three interlocking theoretical contributions. First, and most consequentially, it supplies a missing microfoundational mechanism that bridges the geopolitics-of-data literature in international relations with the privacy decision-making literature in information systems—two productive traditions that have, until now, talked past one another. The geopolitics-of-data scholarship (Aaronson, 2023; Bradford, 2023; Farrell and Newman, 2019, 2023) has theorized how states weaponize asymmetric chokepoints and convert digital infrastructure into instruments of statecraft, but it has remained state-centric and largely silent on how citizens—who bear the diffuse costs of such policies—come to support them. Conversely, the privacy calculus tradition (Dinev and Hart, 2006; Smith et al., 2011) and its recent extensions to generative AI services (Dahabiyeh et al., 2026; Mazhar et al., 2026; Zhou and Tang, 2025) have produced sophisticated accounts of individual-level trade-offs, but they model the relevant choice as a transaction at the individual–service interface and lack analytic purchase on collective-good provisions whose locus of risk is geopolitical. By specifying PFDD as a cognitive bridge—a perceptual construal of the country’s reliance on externally controlled platforms, infrastructures, and AI systems—and demonstrating that this construal translates into individual policy preference through dual pathways with non-trivial effect sizes, we connect the two literatures at the level at which they must be connected: not at the policy outcome but at the cognitive process that links structural condition to individual judgment. This reframes citizen support for digital sovereignty as a perceptual phenomenon explicable within a unified framework rather than as an artifact of elite cues or aggregate national interest, and supplies a portable construct that future scholarship can deploy in other jurisdictions and other domains of infrastructural dependence.

Second, the study refines the dual-process logic of policy attitude formation by demonstrating that risk-oriented and opportunity-oriented construals operate as complementary rather than substitutable mediators. The privacy paradox literature (Acquisti et al., 2015; Kokolakis, 2017) has long observed that risk awareness and behavioral choice do not map cleanly onto one another, and has typically treated risk and benefit as competing terms in a single calculus. Our results complicate this picture: PCDR and TCDO do not cancel one another out but reinforce one another in the same direction with respect to CDPA. A citizen who registers heightened foreign dependency simultaneously appraises cross-border flows as riskier and withdraws tolerance for the openness those flows entail, and both effects converge on greater willingness to bear personal costs for protection. This complementary-mediation pattern (Zhao et al., 2010) suggests that costly data protection support is not a simple risk-driven response but a structurally integrated judgment in which the same perceptual input is processed along two evaluative dimensions whose outputs sum rather than offset. This finding extends the dual-process tradition into the geopolitical domain and provides a template for distinguishing complementary from competing mediators in future studies of costly collective-good support.

Third, and most distinctively, the study reframes the longstanding ‘substitute versus complement’ debate over Data Self-Efficacy by showing that capability does not operate uniformly across evaluative construals, and that the pattern that emerges is neither a uniform substitute account nor a uniform complement account but a third configuration we term the Enlightenment-Vigilance hypothesis. Digital divide research has predicted that capable citizens manage risk privately and therefore disengage from collective protection (the substitute account; Hargittai, 2010), while civic-engagement scholarship has predicted that capable citizens are more attentive to systemic conditions and more supportive of collective action (the complement account; Park, 2013; van Deursen and Helsper, 2018). Our results support the complement account on the risk-oriented pathway—H7 confirms that DSE strengthens the PFDD→PCDR translation—but they decisively reject the original substitute-style prediction we ourselves had advanced for the openness pathway. Instead of preserving their tolerance for openness in the face of dependency, capable citizens withdraw it more sharply (*β* = −0.273, *p* < 0.001). The implication is theoretically consequential. Capability does not produce a discriminating profile in which the citizen distinguishes ‘legitimate openness benefits’ from ‘illegitimate dependency risks’ and thereby preserves the former while sanctioning the latter; rather, it produces an integrated vigilance profile in which the same registered dependency triggers a coordinated downward revision of the legitimacy of cross-border openness itself. Capable citizens, in short, do not merely perceive more risk—they update their normative stance toward the openness regime more decisively. This is, we suggest, the cognitive signature of a politically engaged digital public that has internalized the geopolitical recoding of data: openness is not refused on grounds of incapacity to manage it, but on grounds of an articulated judgment that openness under conditions of foreign dependency is itself the structural problem. The Enlightenment-Vigilance configuration thus speaks directly to the broader debate over whether digital capability empowers political voice or substitutes for it, by showing that, in the geopolitical-data domain, capability empowers a unified rather than discriminating policy stance.

### 5.3 Policy and practical implications

The findings carry implications for digital-sovereignty policymakers, regulators, and platform actors operating in the geopolitical-data environment. First, the dual-pathway result suggests that public communication strategies built solely on risk amplification—a common pattern in cybersecurity and data-localization advocacy—engage only one of two evaluative pathways through which support is generated. Communication that also articulates the normative concerns at stake in cross-border openness under conditions of foreign dependency would activate the TCDO pathway and, by virtue of the complementary-mediation structure documented here, would generate convergent rather than redundant support. Policymakers should treat the two pathways as separable communicative levers rather than substitutes.

Second, the Enlightenment-Vigilance finding reframes the strategic posture appropriate toward digitally capable publics. A common policy reflex assumes that high-capability citizens will either tolerate cross-border openness on instrumental grounds or manage their own exposure privately, and that mass-protective measures must therefore be designed around lower-capability constituencies. Our data suggest the opposite: it is precisely the digitally capable segment that, when foreign dependency is salient, will most decisively support costly collective protection and most decisively withdraw legitimacy from cross-border openness. Policy designs that anticipate resistance from this segment, or that depend on its tolerance to sustain open-flow regimes, are likely to misread the underlying coalition. Conversely, sovereignty-oriented measures—data localization requirements, foreign-platform restrictions, sovereign AI infrastructure—are likely to draw their most articulate political support from this segment rather than encounter its opposition.

Third, for platform actors whose business models depend on cross-border data flows, the findings imply that capability-building campaigns aimed at end-users do not function as a benign substitute for substantive sovereignty concessions. The implicit assumption—that ‘educated’ users will tolerate cross-border flows because they understand the trade-offs—is not supported. Capability operates as an amplifier of the geopolitical recoding of data, not as a mediator that dilutes it. Platforms seeking to retain regulatory legitimacy in jurisdictions where foreign-dependency salience is rising will need to address the construal itself—through verifiable localization, transparency of cross-border transfers, and structural concessions to sovereignty claims—rather than rely on user sophistication to absorb the political pressure.

Finally, the findings speak to the legitimacy basis of data-sovereignty regulation. Because these measures are the product of administrative action that imposes non-trivial costs on citizens, their durability rests not only on formal-legal authority but on what the governance literature terms output legitimacy—the problem-solving acceptability of a policy to those it binds (Scharpf, 1999). Evidence that a specifiable public will bear personal costs for collective protection furnishes regulators with precisely the kind of empirical input that ex ante regulatory impact assessment is designed to capture, and locates the demand for protection in the citizenry rather than in elite preference alone. We are careful to note, however, that measured public acceptance bears on the legitimacy of a policy’s ends and not on its legal validity or on the safeguards owed to citizens as rights-bearers—above all the protection of privacy and personal data against arbitrary administrative action—and that durable sovereignty regimes will ultimately depend on coordinated cross-border governance of the kind now under debate at the international level (Mone and Mitharwal, 2024).

### 5.4 Limitations

Four limitations should be borne in mind when interpreting these findings. First, the empirical base is a single-country cross-section. South Korea offers a theoretically motivated site for studying the geopolitics-of-data construal—it occupies a structurally exposed position vis-à-vis U.S. and Chinese digital infrastructures, and its public discourse has been actively recoding data flows in sovereignty terms—but generalizability across jurisdictions is not warranted on the basis of this design alone. South Korea’s data-protection regime is moreover institutionally specific—organized around the Personal Information Protection Act (PIPA) and its dedicated enforcement architecture—so that transposing these findings across borders is a matter for comparative legal-institutional analysis rather than direct replication of the survey. The Enlightenment-Vigilance pattern, in particular, may be conditioned on the political salience of digital sovereignty in the surrounding discourse; in jurisdictions where this salience is lower, the moderating role of DSE may attenuate or even reverse. Second, the cross-sectional design recovers correlational structure consistent with the hypothesized causal model, but does not adjudicate causal direction. PFDD, PCDR, TCDO, and CDPA are plausibly mutually constitutive over time, and reverse and reciprocal effects cannot be ruled out without temporal separation or experimental manipulation of PFDD salience. Third, although we draw on validated multi-item scales for all latent constructs, all variables are measured via self-report from a single instrument, and unobserved heterogeneity in respondents’ background political orientations may shape both PFDD and CDPA simultaneously. We treat the dual-pathway and Enlightenment-Vigilance patterns as robust given the model fit and the directional consistency of the effects, but the causal claim should be read as conditional on these design features. Fourth, the realized sample skews toward more highly educated respondents. Because educational attainment is empirically entangled with Data Self-Efficacy, this compositional feature may compress variance at the lower end of the DSE distribution and lead the Enlightenment-Vigilance interaction to be estimated on a relatively capability-rich sample; the moderating estimates should accordingly be read as most secure within that stratum and as provisional for populations of markedly lower digital capability.

### 5.5 Future research

Several research directions follow naturally from these contributions and limitations. First, the cognitive-bridge logic invites comparative replication across jurisdictions that differ in the political salience of digital sovereignty. Replicating the model in settings with high salience (e.g., EU member states under the Brussels-effect regulatory regime, or Indo-Pacific jurisdictions exposed to U.S.–China infrastructural competition) and in settings with low salience (jurisdictions in which data flows have not been politically recoded) would allow PFDD’s translational power to be tested as a function of the surrounding discourse. Second, the Enlightenment-Vigilance configuration calls for designs capable of disentangling its constituent mechanisms. Does digital capability operate through enhanced exposure to sovereignty-framed media content, through heightened domain-specific political knowledge, or through a generalized stance of structural attentiveness independent of issue-specific knowledge? An experimental design that manipulates information environment while measuring capability would adjudicate among these mechanisms and either generalize or restrict the present account.

Third, longitudinal designs are needed to trace the temporal dynamics of the dual-pathway translation. Geopolitical shocks—data-related sanctions, infrastructure incidents, high-profile cross-border data scandals—provide natural-experiment opportunities to observe whether PFDD shifts first and CDPA follows, or whether both update in tandem with the underlying discursive shift. A panel design covering such shocks would discriminate among competing temporal orderings. Fourth, the present framework treats CDPA as a unidimensional outcome capturing willingness to bear personal costs for collective protection. Future work should disaggregate the cost dimension: are citizens equally willing to bear convenience costs, monetary costs, and freedom-of-information costs, or do the dual pathways translate differentially into different cost types? Disaggregating the outcome would clarify which specific policy designs—data-localization mandates, sovereign-cloud subsidies, foreign-platform restrictions—are most likely to draw public support, and under what configurations of PFDD, DSE, and political salience.

Fifth, the account developed here invites integration with the analysis of administrative and public law. The present framework explains why citizens come to support costly protection, but is deliberately silent on the questions a legal analysis would foreground: the legal basis on which sovereignty measures are authorized, the differing administrative instruments—general regulations, individual decisions, public contracts—through which they are enacted, and the control and accountability mechanisms that render their implementation reviewable. Bringing the perceptual account into contact with the general principles of good administration would allow the demonstrated structure of public acceptance to inform, rather than substitute for, the assessment of whether and how such policies may legitimately be implemented—an agenda for which PFDD offers a portable measurement entry point across regulatory domains.

Taken together, these directions outline a research agenda in which PFDD operates as a portable cognitive construct, dual-pathway translation as a transferable analytic template, and the Enlightenment-Vigilance configuration as a falsifiable hypothesis to be tested across contexts. We hope this study contributes a usable foundation on which such an agenda can be built, and that the framework developed here proves serviceable for scholars working at the intersection of international political economy, digital sovereignty policy, and the psychology of collective-good support in the geopolitical-data era.

## Data Availability

The datasets generated and analyzed for this study are publicly available in the Zenodo repository at https://doi.org/10.5281/zenodo.20356521.

## Footnotes

1
https://www.embrain.com/eng/
