# Supplementary material for: The Price of digital sovereignty: how Data Self-Efficacy and perceived foreign data dependency drive citizen acceptance of costly cross-border data protection
Source: Front Psychol. 2026 Oct 2;17:1889373. doi: 10.3389/fpsyg.2026.1889373 (PMC13672562; doi:10.3389/fpsyg.2026.1889373)
Supplement: Supplementary file 1 [file Supplementary_file_1.docx]

**SUPPLEMENTARY MATERIAL**

**Appendix A. Measurement Items**

Article 1889373 · Frontiers in Psychology

All research-construct items were measured on a seven-point Likert scale ranging from 1 (strongly disagree) to 7 (strongly agree) and are presented here in English translation. The instrument was developed in Chinese and administered in Korean (see Section 3.2.2). Demographic items are described in Section 3.2.1.

| **Construct** | **Code** | **Item (English translation)** | **Source** |
| --- | --- | --- | --- |
| Perceived Foreign Data Dependency (PFDD) | PFDD1 | I believe our country still relies on foreign cloud services for data storage. | Self-developed; grounded in Farrell & Newman (2019) |
|  | PFDD2 | I believe much of our country's critical data is processed or managed by foreign platforms. |  |
|  | PFDD3 | I believe our country's data infrastructure depends to a considerable extent on foreign firms. |  |
|  | PFDD4 | I believe our country lacks autonomy in cross-border data processing. |  |
|  | PFDD5 | I believe foreign digital platforms play an important role in our country's data operations. |  |
| Perceived Cross-Border Data Risk (PCDR) | PCDR1 | When my data are transferred to other countries, I worry about their security. | Adapted from Dinev & Hart (2004, 2006) |
|  | PCDR2 | I believe cross-border data flows may increase the risk of data being misused. |  |
|  | PCDR3 | I worry that data stored abroad may be accessed without authorization. |  |
|  | PCDR4 | When data are transferred to other countries, I believe it is difficult to effectively regulate how they are used. |  |
|  | PCDR5 | Overall, I believe cross-border data flows entail a considerable degree of risk. |  |
| Tolerance for Cross-Border Data Openness (TCDO) | TCDO1 | I can accept data flowing between different countries. | Self-developed; informed by Hainmueller & Hiscox (2006, 2010) |
|  | TCDO2 | Even when risks exist, I can tolerate cross-border data transfers. |  |
|  | TCDO3 | I consider it acceptable for foreign platforms to process our country's data. |  |
|  | TCDO4 | I regard cross-border data flows as an understandable feature of the digital economy. |  |
|  | TCDO5 | Overall, I hold a relatively tolerant attitude toward cross-border data openness. |  |
| Costly Data Protection Acceptance (CDPA) | CDPA1 | Even if it reduces the convenience of some digital services, I am willing to accept stricter data protection measures. | Self-developed; grounded in Funk (2000) and Stadelmann-Steffen & Dermont (2018) |
|  | CDPA2 | Even if cross-border data restrictions raise service prices, I consider this acceptable. |  |
|  | CDPA3 | Even if restricting cross-border data affects my work or studies, I still support these measures. |  |
|  | CDPA4 | Even if data protection measures reduce the functionality of some services, I am willing to accept them. |  |
|  | CDPA5 | Even if data protection brings inconvenience, I believe these measures are worthwhile. |  |
| Data Self-Efficacy (DSE) | DSE1 | I am confident in my ability to protect my own data from misuse. | Adapted from Eastin & LaRose (2000) |
|  | DSE2 | I know how to manage data-related privacy settings. |  |
|  | DSE3 | I am able to judge which data uses are safe. |  |
|  | DSE4 | When facing data risks, I know how to respond. |  |
|  | DSE5 | Overall, I am confident in my ability to manage my own data security. |  |
